# Policy responses to COVID-19: lessons for the global trade and investment regime

**DOI:** 10.1186/s12992-023-00961-6

**Published:** 2023-09-01

**Authors:** Rachel Thrasher, Warren Kaplan, Veronika J. Wirtz, Louise Clear, Shiva Priya Bodduluri, Sandra Polaski

**Affiliations:** 1 Global Development Policy Center, Boston University, 53 Bay State Road, Boston, MA 02215 USA; 2 Global Health, Boston University School of Public Health, 715 Albany Street, Boston, MA 02118 USA; 3 Department of Global Health, Boston University School of Public Health, 715 Albany Street, Boston, MA 02118 USA; 4 Boston University School of Public Health, 715 Albany Street, Boston, MA 02118 USA; 5 Global Development Policy Center, Boston University, 53 Bay State Road, Boston, MA 02215 USA

**Keywords:** COVID-19, Trade and investment rules, Pandemic policies

## Abstract

**Background:**

During the past two years, the COVID-19 pandemic has cost millions of lives around the globe, caused major morbidity and provoked widespread economic and social disruption. In response, governments have enacted policies to mitigate the impacts of the pandemic. This research focuses in on policies aimed at increasing access to essential health products and services by comparing them to the global rules governing trade, investment and intellectual property. We have assessed whether these rules have or could have constrained countries in responding to this and future crises. The study identifies the nature and scope of the trade-related health sector policies implemented by our sample group of countries, selected because of their systemic significance: the United States, Germany, France, China, South Africa and India. Each policy is placed into one of five broad categories covered by trade and investment rules so that we could assess their consistency with those rules.

**Results:**

We found, among other things, that the types of trade-related health measures were quite diverse. The high-income countries in our study were the most active in the policy space and tended to rely on subsidies-based measures while the middle-income countries relied more heavily on export and import measures. Policies directly relevant to intellectual property protection were virtually non-existent. When evaluating the implemented policies against the global trade and investment rules, we found potential constraints under five different types of rules: those governing subsidies, import and export trade barriers, investment measures, government procurement and trade-related intellectual property.

**Conclusions:**

Given the tension between the global rules and the practices of policymaking during the pandemic, we conclude that the tension must be resolved in favor of governments making policy rather than relying on existing exceptions or pushing national governments to comply more exactly with the rules. Although the pandemic itself does not respect national borders, governance still generally occurs at the national level because national governments are often the only entities with both the legal authority and the practical ability to respond.

**Supplementary Information:**

The online version contains supplementary material available at 10.1186/s12992-023-00961-6.

## Background

During the past two years, The COVID-19 pandemic has cost millions of lives around the globe, caused major morbidity and provoked widespread economic and social disruption [1].

In response, governments have enacted policies to mitigate the impacts of the pandemic, including those aimed at personal protection and health, those offering financial stability in the face of the economic impacts, and those focused on producing and deploying key COVID-19 products. This present research principally considers policies of the third type—aimed at increasing access to essential health products and services—in the context of the global rules governing trade, investment, and intellectual property. We wish to assess whether these rules have constrained, or could constrain, countries in responding to this and future crises.

Many global trade and investment rules reflect a definite preference for policies that allow goods and capital to flow freely across the globe. By contrast, global intellectual property (IP) rules require countries to protect the rights of those holding the protected knowledge and ensure that others do not use that knowledge without permission.^1^ For many countries, this tension between trade and investment policies and IP policies (the latter which countries may be reluctant to change) becomes exacerbated during an acute crisis such as the pandemic. The actions taken to mitigate the impact of the COVID-19 pandemic present an important opportunity to identify aspects of current trade rules that could impede appropriate crisis responses and to address areas where those rules and corresponding institutions should be reformed.^2^

This paper draws on existing scholarly literature to explore how to resolve the tension between rule-based constraints and trends in pandemic policymaking. We conclude that governments need policy space for experimentation so that they can seek to meet the needs of their populations in a crisis, without facing an international dispute as a result.

## Methods

The study begins by identifying the nature and scope of the trade-related health sector policies implemented by a sample group of countries during from March 1, 2020 to August 31, 2021. It includes six large countries whose actions could be systemically significant given their economic power exercised through health product supply and demand, their roles in the global conversation around access to medicines and geographical diversity: the United States, Germany, France, China, South Africa and India. Concretely, the authors selected countries known to have taken action of various sorts to mitigate the effects of the pandemic. The United States (US) and the European Union (EU) were the major suppliers for global vaccines from the beginning. China, likewise, was a major supplier for low- and middle-income countries, and India is well-known for its pharmaceutical industry and production capacity. Together with India, South Africa led the charge to call for a Waiver to the Agreement on Trade-Related Intellectual Property Rights (TRIPS Agreement) and is a regional leader in pharmaceutical production. According to the World Bank classification, the US, Germany and France are classified as ‘high-income’ while China and South Africa are considered “upper-middle income” and India, “lower-middle” [2].

This research draws primarily from the Global Trade Alert (GTA) database which seeks to document all trade-related interventions implemented by states during this period. For each country it tallies the total number of state acts identified as “harmful/discriminating” (“red”), “likely harmful/discriminating” (“amber”) or “liberalizing” ("green") toward trading partners. A second source of data is the World Intellectual Property Organization’s (WIPO) COVID-19 IP Policy Tracker which catalogued all changes to IP laws made during the study period. We then place each of the intervention types documented in these two databases into five broad categories covered by the trade and investment regime: subsidies, trade measures, investment measures, government procurement and intellectual property measures, and identify the main global rule-based constraints which could prove obstacles to these policies.

This study focused primarily on policies aimed at increasing production of or access to key COVID-19 related products, such as health technologies, diagnostics, personal protective equipment, treatments and vaccines. We exclude more general policies aimed at alleviating economic distress, as well as policies aimed at domestic behaviors, such as social distancing, mask mandates, stay-at-home orders, school closures, vaccine rollouts and travel restrictions.

### Cataloging policies

We gathered information from various web-based databases that track government intervention during the pandemic. Our search uncovered eight relevant databases (Table 1), which had various lists of pandemic-related policy responses.

**Table 1:** Web-based sources for relevant policy responses

Name	URL	Description	URL
Policy Response to COVID-19	International Monetary Fund (IMF)	Narrative summaries of key economic responses by governments to limit the human and economic impact of the pandemic. Includes 197 economies	https://www.imf.org/en/Topics/imf-and-covid19/Policy-Responses-to-COVID-19
COVID-19 IP Policy Tracker	World Intellectual Property Organization (WIPO)	Information on measures adopted by IP offices in response to the COVID-19 Pandemic (no longer available)	https://www.wipo.int/covid19-policy-tracker/
COVID-19 Temporary Trade Measures	International Trade Centre (ITC)	Catalog and mapping of temporary trade measures (import and export) imposed by countries in response to COVID-19	https://www.macmap.org/covid19
Key policy responses from the OECD	Organisation for Economic Co-operation and Development (OECD)	Comprehensive catalog of all measures in various categories imposed by OECD countries (includes fiscal and monetary, employment and social and health policies)	https://www.oecd.org/coronavirus/en/policy-responses
COVID-19 Government Response Tracker	Blavanik School of Government and Oxford University	Working Paper (regularly updated) with a catalog of government responses to COVID-19 as well as analysis of how those measures correlate to changes in COVID-19 cases, hospitalizations and deaths	https://www.bsg.ox.ac.uk/research/covid-19-government-response-tracker
COVID-19 Financial Response Tracker Visualization	Yale School of Management	Catalog and mapping of individual government economic financial policies introduced or amended to combat the negative effects of the coronavirus outbreak	https://som.yale.edu/centers/program-on-financial-stability/covid-19-tracker
Global Trade Alert (GTA)	Foundation with support of University of St. Gallen, the Max Schmidheiny Foundation and Prof Simon Everett, the St. Gallen Endowment for Prosperity through Trade among others	Comprehensive catalog of all government measures imposed since 2009, identified primarily by type of measure and whether it is trade “liberalizing” or “harmful”	https://www.globaltradealert.org/
COVID-19 Regulatory Measures	Institute of International Finance (IIF)	Catalog of financial and stabilization policies imposed by developed country markets, as well as the IMF and the G20	https://www.iif.com/Research/Download-Data#C19Tracker

After a preliminary assessment of these databases and documents, we decided to use the Global Trade Alert (GTA) database as our primary data source, while relying on the other web-resources as secondary sources that contain information not available in the GTA dataset such as intellectual property policies. This is based on the comprehensive nature of the GTA database, as well as the related information for each government intervention. Each measure is categorized within a specific typology of government intervention and flagged as "red", "amber" or "green", as indicated above, depending its predicted impact on global trade [3].^3^

To further support the decision to rely primarily on the GTA database, we validated the information that was presented in GTA against the other databases mentioned above using the following assessment questions:

- How much and what kinds of overlap in information is there between the GTA and the other databases?
- How easy is it to determine this overlap, if any exists? For instance, do the databases use a variable that can be used to link both databases (e.g. name of the state act)?
- Can the other databases provide complementary information not found in GTA that is relevant to this research? (e.g., additional information about the content, structure or context of a particular policy that allows us to deepen our analysis)

For a random sample of policies, we compared the results of the GTA database with the IMF and the Oxford COVID-19 Government Response Policy Tracker. The validation was performed by comparing start date/month of policy as well as comparing policy information registered in the GTA database with the IMF and Oxford databases. The information was congruent in more than 95% of cases.

The data gathered from the GTA includes measures beginning on March 1, 2020, as indicative of the beginning of government awareness and intervention in response to the pandemic. The database was published on July 31, 2021 and assayed on August 31, 2021 by downloading into Excel.

### Intervention types and the current trade and investment rules

We categorized the 24 GTA-identified intervention types from our study into four broad categories: subsidies, trade measures (tariffs and quantitative restrictions), investment measures and public procurement. These categories align generally with the way that the creators of the GTA group their policy data ([1] capital controls and exchange rate policies, [2] export and import measures, [3] foreign investment measures, [4] labor force migration rules, [5] localization requirements, [6] public procurement, [7] subsidies and state aid, [8] trade defense instruments and [9] other instruments) [4] [4]. We combined export and import measures with trade defense instruments – identified collectively as “trade measures” – because they are governed by treaty provisions covering trade in goods. Localization requirements were grouped with investment measures where they applied to foreign investment generally, which are governed by investment chapters in free trade agreements (FTAs) and standalone international investment agreements (IIAs). Localization requirements which are specifically tied to public procurement rules were put in the public procurement category. Given that the GTA did not capture any changes to IP laws or grants of compulsory licensing, we added policies from the World Intellectual Property Organization’s (WIPO) COVID-19 IP Policy Tracker to make sure we included those policies as well.

Two authors (RDT and SPB) grouped the intervention types from all the data (GTA plus WIPO Tracker) collected now into five categories (1) subsidies, (2) trade measures, (3) investment measures, (4) public procurement and (5) IP policies. based on the types of treaty rules that would govern those policies. Where discrepancies between the data extraction by the two authors occurred, two other authors (WAK and VJW) verified the categorization. When an intervention was labeled differently in sources outside of the GTA we re-categorized it to more specifically reflect the nature of the intervention. For instance, our team chose to categorize US subsidies to private production of vaccines as production subsidies rather than the GTA’s choice to put them into the more amorphous state aid category. Our study did not find any capital controls/exchange rate policies, labor force migration rules or other instruments for the countries and time period covered.

Once categorized, we used a purposive sample of policy acts in each of the five categories for each of the countries based on whether these different policy acts appear in in trade and health policy literature and business/economic reporting. Examples of such policies are India’s licensing requirement for exports of Amphotecerin B, the US, EU and Indian government support for vaccine development and the US, EU and South African airline support measures [5–8]. We then compared policies in each of these five categories with legal constraints present in trade, investment and intellectual property rules as exemplified in the World Trade Organization (WTO) agreements, as well as additional rules present in bilateral and regional free trade agreements (FTAs). We selected 4 key free trade agreements, including provisions from a recent EU Association Agreement (EU-Ukraine) [9], the most recently negotiated US FTA (USMCA) [10], and two distinct regional FTAs from the Asia–Pacific region (the Comprehensive and Progressive Trans-Pacific Partnership Agreement (CPTPP) [11] and the Regional Comprehensive Economic Partnership (RCEP) [12]). The constraints on policymaking made by the WTO and FTA rules is well-established in the literature [13–15]. We relied on that literature to map illustrative policy examples onto the relevant international legal constraints. We note that a given EU policy with regard to France and Germany (See Table 2) was counted separately for each country.

**Table 2:** Total number of distinct policy interventions, categorized by type of policy intervention

Country (in order of nominal GDP per capita) (1)	GDP 2020^a^ (international $)	Total # of interventions enacted (2)	# of health sector interventions (as % of total) (3)	# of health sector subsidies (as % of health sector measures) (4)	# of health sector trade measures (5)	# of health sector investment measures (6)	# of health sector public procurement measures (7)	# of health sector IP measures (8)
USA	59,920	476	70 (14.7%)	49 (70%)	19 (27%)	1 (1%)	1 (1%)	0
Germany	51,451	263	28 (10.3%)	15 (54%)	9 (32%)	3 (11%)	0	1 (4%)
France	42,321	161	39 (23.8%)	26 (67%)	8 (21%)	4 (10%)	0	1 (3%)
China	16,316	32	7 (21.9%)	2 (29%)	5 (71%)	0	0	0
South Africa	12,666	33	5 (15.2%)	3 (60%)	2 (40%)	0	0	0
India	6,166	170	56 (32.9%)	17 (30%)	32 (57%)	3 (5%)	4 (7%)	0

*Sources*: GTA 2021; WIPO 2021 [16]; Authors’ calculation

^a^Gross Domestic Product in international dollars. World Bank in Our World Data (https://ourworldindata.org/grapher/gdp-per-capita-worldbank)

## Results

### General findings: the catalog of policy interventions

We found that the types of trade-related health measures deployed during the pandemic were diverse, ranging from policies aimed at funding or collaborating with pharmaceutical companies, to increasing domestic investment in health sectors, to policies attempting to reduce shortages of essential products by ramping up their domestic manufacturing or preventing their exportation. Moreover, while all the countries in the study were active in implementing a wide array of measures, the high-income countries in our study (US, Germany France) were generally much more likely to take policy action.

Table 2 quantifies the total number of distinct policy interventions, categorized by our five-part taxonomy, focusing especially on the health sector (column 3).^4^ The table shows the most prevalent types of policy interventions by each country (Table 1: columns 4–8). For our sample of countries, subsidy policies (column 4) were the most numerous and among the most diverse, e.g., targeted subsidies to domestic producers, capital injections into private firms, government advance purchase agreements for vaccines and treatments, and others. Tariffs and quantitative restriction policies followed next (column 5), e.g., import and export restrictions, import and export licensing requirements, tariff quotas, and others. Investment measures (column 6) included only a short list of new rules governing foreign direct investment, local sourcing and localization incentives. Public procurement policies (column 7) governing direct government purchases of goods and services were less extensive and included access rules, localization requirements and preference margin incentives.

Policies directly relevant to the health demands of the pandemic that could be constrained by trade policies related to intellectual property, such as those found in the WTO Agreement on Trade-Related Aspects of Intellectual Property (TRIPS agreement) were virtually non-existent. In our sample, only France and Germany each made changes to their compulsory IP licensing procedures to make such licenses easier to issue during the pandemic.

**Fig. 1:**
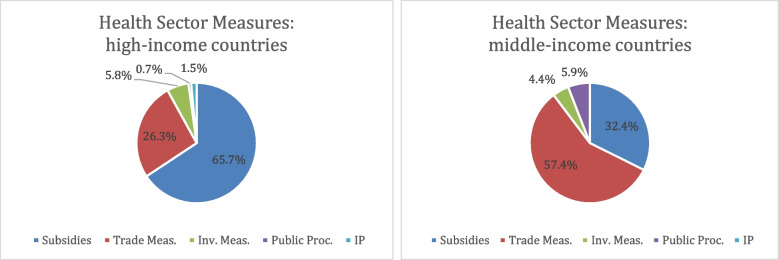
Health sector intervention types according to country income level

As shown by Table 2, the higher the nominal GDP per capita of each country, the higher the number of interventions, except for India. The US dominates the list with 476 distinct total policies as well as with the greatest number of trade-related health sector policies, primarily subsidies. For the US, France and Germany, subsidies were the most common policy tools, while procurement and investment measures were the least common. In contrast, China and India relied relatively more on tariffs and quantitative restrictions than on subsidies (see Fig. 1).^5^ India’s interventions focused heavily on the health sector compared with the other study countries, although these measures still made up a minority of its interventions. China implemented the fewest number of policy interventions during the time period.

Table 3 disaggregates broader intervention types more specifically. Some policy tools are used by most or all countries: state aid or state loans, export licensing requirements, import tariffs and anti-dumping duties. The data show the prevalence of subsidies, especially by our high-income countries. India deployed the greatest number of distinct intervention types, while South Africa used the fewest. It is also notable that the policy types deployed by France and Germany are the most similar to one another, likely reflecting the fact that the pandemic response was partly shaped at the level of the regional level in the EU.

**Table 3:** Intervention types by country

Broad intervention category	Intervention type	USA	Germany	France	China	SA	India
Subsidies	Capital injection (e.g., bailouts)			2			
	Financial grant	18		8			3
	Interest payment subsidy						1
	Loan guarantee		4	4			
	Production subsidy	1					7
	State aid	28					3
	State loan	2	9	11	1	3	
	Tax or social insurance relief	1		1	1		1
	Trade finance		2				2
Tariffs and quantitative restrictions	Anti-dumping duty	4	2	2	1		19
	Anti-subsidy duty	3			1		1
	Export ban						1
	Export licensing requirement	1	4	4	1	2	4
	Export quota						1
	Export-related NTM	10					1
	Import ban						4
	Import licensing requirement				1		
	Import tariff	1	1	1	1		1
	Import tariff quota		2	1			
Investment measures	FDI: Entry and ownership rule		1	2			
	FDI: Treatment and operations, nes	1					
	Local sourcing						3
	Local operations		2	2			
Public procurement	Public procurement localization	1					4
Intellectual property protection	Compulsory licensing procedures		1	1			
**TOTAL**		70	27	38	7	5	56

*Source*: GTA 2021; WIPO 2021 [16]; Authors’ calculation

Table 4 provides examples of the policy typology exemplified by information found in the GTA and WIPO databases (column 1). The relevant WTO rule regarding the specific policy type is found in column 2 and an example of the WTO rule as provided in particular free trade agreements is in column 3.

**Table 4:** Illustrative typology of policies and relevant provisions

Policy Type and Examples	WTO Rule (citation)	FTA rule (example)
**Subsidies:** ➢ US, EU, India and China’s government support for vaccine development (Moderna, Pfizer/BioNTech, Covaxin, Sinovac and others) ➢ South Africa's support for firms producing COVID-19 supplies ➢ India's subsidies for firms manufacturing medical devices ➢ Various countries’ subsidies for acquisition of diagnostic equipment	Specific subsidies are actionable if they cause injury or result in serious prejudice to foreign competitors (SCM Arts. 1 (definition of subsidies), 5 (adverse effects of ‘specific’ subsidies), 6.3 (serious prejudice))	None beyond the WTO rules
**Import/Export Restrictions:** ➢ South Africa's and India's new export licensing measures ➢ India's export ban on vaccines and Germany's export ban on PPE	No quantitative restrictions on trade – import or export, except where there are “critical shortages of essential goods” (GATT Article XI)	EU-Ukraine Art. 271 USMCA Art. 2.11 CPTPP Art. 2.10 (adopting and incorporating GATT Article XI) RCEP Art. 2.17 (adopting and incorporating GATT Art. XI)
**Investment Measures:** ➢ India's local content requirement on medical coveralls ➢ France and Germany's new foreign investment screening in health and biotech sectors ➢ EU advanced purchase agreements with localization requirements (AstraZeneca & Curevac)	No measures which require foreign investors to use local content or export a certain % of their goods (TRIMS Art. 2, Annex)	All including right of establishment: EU-Ukraine Art. 88 (national treatment) USMCA 14.4 (national treatment), 14.10 (performance requirements) CPTPP 19.4 (national treatment), 19.10 (performance requirements) RCEP Arts. 10.3 (national treatment), 10.6 (performance requirements)
**Government Procurement:** ➢ India's new local procurement rules for medical device producers selling to the government ➢ US advanced purchase of vaccines ➢ US SEPIR initiative ➢ EU advanced purchase agreements with localization requirements (AstraZeneca & Curevac)	Requires non-discrimination and transparency in government purchasing and contracting decisions among parties to the agreement (GPA Art. IV)	EU-Ukraine Art. 151 (non-discrimination and transparency) USMCA Art. 13.4 (non-discrimination and transparency) CPTPP Art. 15.4 (non-discrimination, and transparency) RCEP Art. 16.4 (transparency only)
**IP/Compulsory Licensing:** ➢ Germany and France's modification of domestic CL rules	TRIPS Agreement, Article 31, 31bis	EU-Ukraine Art. 219 (reaffirming the Doha Declaration on TRIPS and Public Health) USMCA Art. 20.6 (affirming commitment to Doha Declaration) CPTPP Art. 18.41 (incorporates TRIPS by reference) RCEP Art. 11.39 (incorporates TRIPS Art. 31, 31bis by reference)

*Source*: Authors’ analysis

## Discussion

Our findings show that high-income countries in our study deployed extensive financial resources and subsidies in addition to tariffs, trade constraints and in-kind measures. Middle-income countries in our study, especially China and India, relied more on non-financial measures like export licensing and quantitative restrictions. In the following discussion we map the findings of policy interventions onto the rule-based constraints and discuss the implications of trade and investment treaties for policymaking.

### Mapping policies onto rule-based constraints

What is assessed by the GTA researchers and adopted by the authors, is that these “red” policy interventions have a distorting or negative impact on trade or the flow of investment, and thus are suspect under the trade and investment rules, which prefer liberalized trade and free market economies. Trade and investment treaties, which make up a de facto system of global rules, oversee the process, amount and economic impact of government subsidies as well as (to a lesser degree) public procurement practices. The treaties establish a ceiling for tariffs and a floor for IP protection levels. They place limits on domestic policies governing foreign investment and outright prohibit the use of quantitative restrictions.

It is not self-evident, however, that just because these measures may violate trade and investment rules, they are necessarily bad or undesirable policies for countries to put in place. What follows is a discussion of the role of the global trade and investment rules, how they may have constrained or be constraining domestic policymaking during a pandemic, and what, if anything, needs to be done.

1. Subsidies
  Subsidies were the most prevalent type of policy intervention found in our study. The WTO’s Agreement on Subsidies and Countervailing Measures (SCM) lays down strictures on government support of industries, though it does not prohibit them outright [17]. Subsidies targeting a specific firm, sector or geographic area (“specific” subsidies) can be subject to legal action, either through a WTO dispute or unilateral trade remedies,^6^ provided the complaining country can prove injury to their domestic industry, serious prejudice to their economic interests in other markets (Art. 6.3), or (in a small number of cases) “nullification or impairment” of their expected benefits under the suite of WTO Agreements (Art. 5). Rules governing subsidies are not as common in FTAs. The EU, however, has an extensive and strict set of state aid rules that constrains member states’ subsidies and related policies. In the early days of the pandemic, the EU introduced a “State Aid Temporary Framework” which suspended certain state aid rules to allow member states to respond with targeted support of pandemic countermeasures [18]. As such, subsidies introduced during that time would not contravene EU rules, unless it continued past the expiry date of June 30, 2022.
  In this context, the US financial support to Moderna for research and development on the mRNA vaccine platform, as well as the European Investment Bank’s loan to the German company BioNTech for the same would undoubtedly be “specific” subsidies under the WTO definition (See Table 2, under Germany as a ‘loan guarantee’). State support for vaccine development was not limited to high income countries. In fact, India’s subsidies for domestic vaccine development and production of Covaxin and China’s parallel support for Sinovac would likely raise the same concerns as “specific” subsidies under the SCM. Outside of the vaccine sector, South Africa provided substantial financial support for firms producing COVID-19 supplies and India poured production subsidies into the manufacturing of medical devices. Although none of these subsidies would be outright prohibited under the WTO, they could be challenged by a fellow WTO member whose competing industry was negatively impacted.
  During this pandemic, at a time when all countries acknowledged the necessity of government support and tried novel measures to mitigate the pandemic impacts, the likelihood of WTO disputes seems low given the risks of retaliation.. Nonetheless, states have demonstrated in other contexts, that they are ready and willing to engage in tit-for-tat disputes at the WTO (US-Softwood Lumber VI [19, 20], EC and certain member States – Large Civil Aircraft [21]; India-Solar [22], US – Renewable Energy [23]). As the urgency of the pandemic begins to wane and existing firms seek to consolidate or expand their share of the market, WTO disputes and domestic investigations into subsidies and countervailing measures are likely to make an appearance. In particular, countries wishing to support their nascent pharmaceutical industries will find themselves constrained by the rules preventing them from causing injury to domestic incumbents and industries in other countries. The more successful they are in launching new or expanded domestic industries, the more likely they will be mired in costly disputes.

2. Tariffs and quantitative restrictions
  The second most prevalent interventions were tariffs and quantitative restrictions. While tariffs are generally permitted under the General Agreement on Tariffs and Trade (GATT), though bound to a ceiling determined by each Member country’s schedule (GATT Art. II), non-tariff barriers are largely prohibited (GATT Art. XI:1) [24]. There remains a carve out allowing export restrictions “temporarily applied to prevent or relieve critical shortages… of essential products” (GATT Art. XI:2), but the exact contours of that exception have not been fully tested. Regional and bilateral free trade agreements (FTAs) also contain prohibitions on new non-tariff barriers to trade that tend to mirror both the rules and the exceptions of Article XI (e.g., USMCA Art. 2.11) [10]. General and security exceptions may apply in limited ways to these measures as well and are discussed below.
  Export barriers were a common policy approach to address the scarcity of supply of key COVID-19 products (Table 3). India and South Africa introduced new export licensing requirements for COVID-19 health products. Shortly after the pandemic began, Germany imposed an outright ban on exporting personal protective equipment (PPE) and India addressed a vaccine shortage by introducing a rule of “compulsory domestic sale” for vaccines (effectively an export ban). In each instance, these policies would almost certainly fall under the general prohibition on non-tariff barriers (XI:1), although they might well qualify for the exception for relieving critical shortages in essential products (XI:2), as long as they are only temporary.

3. Investment measures
  Some of the most controversial rules of the global trade regime are those governing treatment of foreign investors and their investments. Under the WTO, the Agreement on Trade-Related Investment Measures (TRIMs agreement) requires that the GATT standard of national treatment (GATT Art. III) and prohibition on new non-tariff barriers (GATT Art. XI) applies to both trade and investment measures (TRIMs Art. 2) [25]. Thus, any measure is likely inconsistent with TRIMS if it shapes the investment environment to restrict imports or exports or prefers domestic to imported products.
  Suspect health sector investment measures under TRIMs include India’s imposition of local content requirements on medical coveralls, German and French introduction of new foreign investment screening in the health and biotech sectors and the EU’s localization requirement for firms negotiating advanced purchase agreements, including AstraZeneca and Curevac. Under the TRIMs Agreement, local content requirements are specifically prohibited measures and localization requirements are likely to have a similar discriminatory impact on the use of imported vs. domestic products.
  IIAs often have much deeper, more specific and more enforceable commitments than those at the multilateral level, [26]. While the TRIMs Agreement applies only to investment measures related to trade in goods, the protections in IIAs typically apply to all sectors where foreign investors are present. In addition, IP is often included as a protected investment, [27]. Furthermore, a prohibition on performance requirements for investors, including localization requirements, limits the range of policy tools at a government’s disposal for managing crises even more [28].
  Finally, the investor-state dispute settlement (ISDS) mechanism prevalent in IIAs provides a unique opportunity in international law for investors to sue national governments in private arbitrations for government regulations claimed to interfere with the value of their investments. Many have written to critique this system [29–31]. We note that allowing private stakeholders to sue states outside of their domestic courts removes the ordinary checks and balances of state-to-state dispute settlement mechanisms based on potentially mitigating diplomatic or public welfare considerations [32–34]. During or after the pandemic, private firms (including pharmaceutical companies) may choose to bring an investor-state claim directly against a country if their in-country investments operations are claimed to have been undermined by policy changes. As discussed below, that could include changes that diminish the expected monetized return on a patent or copyright.

4. Public procurement
  The rules that govern government (or public) procurement practices are somewhat narrower in scope and application. The Government Procurement Agreement (GPA) is a plurilateral agreement within the WTO, meaning that its commitments apply only to its 48 member states (and others that join in the future) [35]. The general rules require members to not discriminate against the products, services and firms of fellow members in their procurement decisions (GPA Art. IV.1, IV.4). The US, for example, has not made any procurement commitments with respect to state or local governments, so that those sub-national governments may make their own decisions about whether to comply with GPA purchasing rules [36]. On the other hand, the US has included a number of agencies that purchase medical supplies and pharmaceuticals for the government in its procurement commitments [37].
  As mentioned, the GPA is plurilateral so that the US does not have to extend the same treatment to China (for example) as it does to fellow GPA-member EU states (e.g., Germany, France). Outside of the WTO, however, countries may have accepted more in-depth procurement commitments – including by covering more sectors under the non-discrimination and transparency rules. The US eliminated government procurement commitments with Canada in the process of renegotiating the North American Free Trade Agreement, reverting to the (less demanding) GPA rules in the new USMCA, although US procurement commitments with Mexico, which is not a GPA member, still stand in the new agreement [37].
  Given the scope of these rules, the EU’s advance purchase agreements with Curevac and Astrazeneca, and the US advance purchase agreements with vaccine suppliers would be covered by the GPA, as well as the US’ new Strategic Active Pharmaceutical Ingredients Reserve (SAPIR)—an effort to increase the domestic manufacturing of essential pharmaceutical inputs. The US, however, is not likely to face a WTO challenge on that basis since it strategically funnels purchasing through state and local governments not covered by the GPA [36].
  India likewise introduced procurement measures. The government required medical device producers supplying directly to government bodies to rely on local inputs for production. These measures are similar to local content requirements generally – which are prohibited under WTO and FTA rules, as well as under the non-discrimination provision of the WTO’s GPA. In the narrow context of procurement, however, since India is not yet a party to the GPA, it would not be bound by those rules. If India were to sign onto the CPTPP, non-discrimination rules in their procurement practices would have to be introduced, whereas RCEP does not require the same standard.

5. Intellectual property protection
  Perhaps an area that has received the most publicity during the pandemic is the role of global intellectual property rules in promoting innovation or limiting access to medicines. The TRIPS agreement provides a well-known baseline of protection, including 20-year pharmaceutical patents (TRIPS Art. 27), with limited exceptions for health emergencies (TRIPS Arts. 30, 31), which are circumscribed by complex rules on domestic efforts to issue compulsory licenses to produce products that are scarce or unaffordable (TRIPS Art. 31) [38]. The rules potentially allow a member state without production capacity to request another country to produce a patented product for its use (TRIPS Art 31*bis*) but this has not been widely used [39, 40]. FTAs have introduced “TRIPS-plus” standards which, in practice, extend patent terms, protect clinical trial and other data for longer periods of time and interfere with marketing approvals for generics [41, 42]. Moreover, many international investment treaties include intellectual property as a covered investment, thus subject to protection and, as mentioned above, enforcement through ISDS.
  While countries have been willing to flout international trade commitments in the current crisis, they have been surprisingly reluctant to ignore global IP rules, and even hesitant to rely on existing flexibilities within the rules, such as compulsory licensing. While Germany and France did make some changes to their compulsory licensing procedures, authorizing new government representatives (in Germany the Federal Ministry of Health and in France, the Prime Minister) to issue compulsory licenses (CLs) during the declared COVID-19 emergency for the benefit of public welfare [16], they did not issue any licenses, and only a small handful of countries did so, with none issued for vaccines [43, 44]. If countries did suspend or modify IP rights, they could be susceptible to claims at the WTO and unilateral pressure from countries and regions like the US and the EU, and perhaps to ISDS claims as well. Rather than ignore the rules, some countries sought an official negotiated waiver of provisions of the TRIPS agreement for COVID-19 related products [45]. Proposed in October of 2020 by India and South Africa [46], negotiations resulted in a ministerial decision that falls far short of the expansive TRIPS waiver initially proposed [47, 48]. The reasons for this are rooted in various complexities discussed in more detail below.

6. Exceptions and consequences
  There are exceptions to many of these trade and investment rules that might make space for non-compliant policies during emergencies. GATT Article XX provides a list of general justifying exceptions such as measures “necessary to protect human, animal or plant life or health” (XX(b)), restrictions on exports to “ensure essential quantities of those materials” (XX(i)) and measures “essential to the acquisition or distribution of products in general or local short supply” (XX(j)) [24]. Although some justifications, especially sub-paragraph (b), could be broadly interpreted – the overarching conditions of the Article’s introductory paragraph require that exceptional measures be applied in a way that does not constitute arbitrary or unjustifiable discrimination or a disguised restriction on trade. The WTO’s Appellate Body has relied heavily on this “chapeau” requirement such that few countries have successfully defended policies under Article XX [49].^7^
  GATT article XXI allows WTO members to take “any action which [they] consider necessary for the protection of [their] essential security interests (iii) taken in time of war or other emergency in international relations” (XXI(b)(iii)). This exception does not include the chapeau requirement of Article XX so it may be interpreted more broadly or flexibly. Similar Article XXI-type language is found in the General Agreement on Trade in Services (GATS Arts [50]. XIV, XIV*bis*), the TRIPS Agreement (TRIPS Art. 73) and the GPA (Art. 11) [35]. Both the TRIMs and SCM Agreements are under the GATT, so these general and security exceptions would apply directly and automatically to them. FTAs also tend to retain the language of general and security exceptions. A previous study by one of the authors found that almost half of all preferential trade agreements notified to the WTO contain an essential security exception similar to that of GATT Article XXI [51].
  In the context of a global pandemic, specific subsidies to support vaccine development and production or export restrictions, for example, could very likely be justified under existing rules and exceptions. Subsidies deemed to be “specific” under the SCM, for example, like India, China, the US and Europe’s support of vaccine development, as well as South Africa and India’s support for the production of other COVID-19 products, could be justifiable under the GATT article XX(b). In the early months of the pandemic, these measures certainly seemed necessary for the protection of human life and health, and once we had vaccines, we saw clearly that their absence would have resulted in even more massive loss of life. Moreover, many countries in the world declared a state of emergency, potentially making available any measures considered “necessary for the protection of their essential security interests” (GATT Art. XXI, GATS Art. XIV*bis*, TRIPS Art. 73, GPA Art. 11). A similar analysis might apply to trade measures aimed at securing a country’s supply of essential products like PPE (Germany) or vaccines (India). As long as a country could demonstrate that the measure is not “applied in a way that it is a disguised restriction on trade or results in arbitrary or unjustifiable discrimination”, the general exceptions are likely to apply, and in times of extreme urgency, the security exceptions might suffice to justify even the most discriminatory of policies.
  Relying on exceptions, however, to defend pandemic policymaking has both short- and long-term shortcomings. Even if a country is able to successfully defend itself against a challenge under the WTO’s dispute settlement mechanism, or in an investor-state dispute, these international cases can take an institutional and financial toll, especially during a time when they are attempting to face a global crisis of public health. If a country’s policies are successfully challenged at the WTO, a few outcomes might result. In the first place, the respondent country will need to bring their measures into compliance with WTO Agreements. This may mean altering their subsidy scheme, removing import or export barriers, or withdrawing a compulsory license, among others. If they are unable or unwilling to take that step, they may face market access consequences sanctioned by the WTO Dispute Settlement Body. Countries that either experience challenges to their policymaking, or watch other countries do so, may be reluctant in the future to engage in policymaking that could disrupt trade, even if it seems important for public health, environmental or other public purposes. Investor-state disputes can have a similar chilling effect and can result in large-scale payouts to investors who claim that the policies had an unforeseen negative impact on the value of their investment.
  At the time of this writing, the WTO’s dispute settlement processes are hamstrung by the lack of an appellate mechanism. The Appellate Body has been without a quorum since December 2019. As a result, a member country may appeal any panel decision that it does not like without any near-term likelihood of having the case resolved. For the time being, then, countries are not likely to face immediate consequences from flouting the WTO rules. Some fear that this will lead to a devolution of the rules-based multilateral trading system into a power-based system like that of the mid-twentieth century. In that latter case, the most powerful countries will be able to ignore rules with impunity, while attempting to enforce them against the less powerful.

### Understanding the tensions and trade-offs

The evidence above makes clear that during the early stages of the COVID-19 pandemic, various countries frequently disregarded or circumvented policy constraints arising from WTO and other trade/investment commitments. As they discovered their need for PPE and for diagnostic tools, treatments and vaccines for COVID-19, many governments became proactive. High-income countries in our study deployed extensive financial resources and subsidies in addition to tariffs, trade constraints and in-kind measures. Middle-income countries in our study, especially China and India, relied more on non-financial measures like export licensing and quantitative restrictions.

What might explain this pattern of policy choices? We speculate that the wealthiest countries preferred subsidies and government procurement to direct trade measures to support domestic production and acquisition of essential goods for two reasons. First, they had the fiscal or monetary space to do so and second, because such measures are considered less directly trade-distorting [52]. Trade measures are more directly trade distortive and are often directly prohibited under the trade rules. Middle-income countries tended to not have the fiscal space of their higher-income counterparts and may have been forced to rely on other policy instruments, regardless of the transgression of trade rules.

Of particular interest was the relative lack of any IP policy changes for our study countries. Given that one of the main externalities of IP protection is decreased access to the protected product for a time, loosening IP rules would have been a natural first step to address shortages of COVID-19 products during the pandemic. Indeed, one of the most well-known flexibilities incorporated into most FTAs with IP commitments is the possibility of issuing a CL, which allows a country to issue a license to a local firm to produce an essential good that is otherwise not available (or unaffordable) to its population. Unfortunately, global IP rules place an onerous procedural burden on countries attempting to issue such a license and as such they are little used [43, 53].

In addition to the legal and institutional complexity of relying on compulsory licensing mechanisms,  the political backlash that countries have faced historically in using, or even threatening to use, CLs, drives their underuse. First of all, in order to issue a CL in accordance with the TRIPS Agreement, each patent involved in the production of a product must be identified. CLs must be issued for each individual patent and sub-manufacturers found for each component of the product or technology. If a country does not have its own manufacturing capacity, it must find another country willing to issue a CL for export. Once more, this must be done for each individual component of the product or technology. This cumbersome and lengthy process renders Articles 31 and 31*bis* highly impractical and virtually meaningless for most circumstances [54]. This difficulty has been extensively documented, before and during the pandemic [55–57].

Secondly, the pharmaceutical industry in the United States has frequently lobbied to punish countries that attempt to issue CLs. The US has repeatedly put pressure on India for is licensing on an expensive cancer drug, claiming that India is “diluting” IP rights and violating the TRIPS Agreement [44]. Private pharmaceutical companies and U.S. lawmakers have even threatened sanctions against India through its Special 301 Report, a trade watch-list of sorts. Colombia faced similar backlash when they took the first steps toward issuing a CL for a leukemia treatment – Glivec [54]. Both the Swiss government and Novartis, the patent holder, argued forcefully that CLs are “tantamount to expropriation” – code for exercising a sort of eminent domain through regulation [54, 58]. More recently, Malaysia attempted to use a CL to increase affordability of a Hepatitis C medication and once more the US, together with its pharmaceutical industry, threatened to wield the power of sanctions through a Special 301 Report [59].

Even during the early months of the pandemic, the pharmaceutical industry was already lobbying for punitive sanctions against countries pursuing these measures – including Hungary, Colombia and Chile [60]. As a result of these and other instances, countries have, understandably, been reluctant to develop more flexible domestic CL policies, and even those that have, are often reluctant to use them [44, 61].

India and South Africa’s October 2020 proposal (and subsequent revision in June of 2021) to waive key provisions of the TRIPS Agreement implicitly recognized that the existing ability to authorize production of essential COVID-19 products was insufficient and inefficient. It covered the full spectrum of IP, data, trade secrets and knowhow that would be required to produce the products. It also recognized that global cooperation is required to effectively increase access to protected health innovation on a scale to address a global pandemic [46, 62].

At the 12^th^ Ministerial Conference of the WTO in June 2022, members adopted a ministerial decision on the TRIPS Agreement, which purportedly addressed the “exceptional circumstances of the COVID-19 pandemic” by “clarify[ying] and waiv[ing]” certain TRIPS provisions [47]. In actuality, the decision does not make any substantive changes to the existing flexibilities available under TRIPS and has been derided as a “disappointing failure” by advocacy groups [63, 64]. The United States took a different promising step when it announced in May 2022 that the National Institutes of Health would be sharing key mRNA technology with the COVID-19 Technology Access Project (C-TAP), the first major producer of mRNA to do so [6]. The World Health Organization (WHO) has begun to collaborate with Afrigen in South Africa to encourage the domestic development and reverse engineering of vaccines beginning with Moderna’s recipe [65] and GAVI is establishing a regional production hub for mRNA vaccines in Africa [66].

### Resolving the tension

Given the tension between global rules and the actual policymaking that took place during the pandemic, how should country leaders resolve it? In broad terms, there are three possible approaches. First, one could argue that nothing need change because the exceptions, described above, should be sufficient to protect legitimate public policymaking from losing a challenge at the WTO or even an investor-state dispute in the context of a global emergency like the COVID-19 pandemic. Second, one could argue that the main problem lies with the overly activist role of states. If they had followed the global rules better, there would have been more global supply and a more equitable distribution for everyone. Third, one could argue that the main problem lies with the rules themselves. Global trade and investment rules should not stand in the way of public policymaking – especially in an acute crisis, but also beyond.

There is a rich literature on the role of exceptions in international jurisprudence [67–69]. The concept of pandemic “exceptionalism” suggests that policymaking trends during a pandemic will not necessarily be normalized outside of that context so that exceptions clauses may be sufficient for the majority of policy interventions undertaken in an emergency [70]. On the other hand, these exceptions are criticized for their insufficiency (e.g., Arato et al. 2020) and this may explain why IP exceptions purportedly designed to address global health emergencies are still going largely unexploited [43].

Should national public health policies and priorities generally take precedence over the current framework of trade, investment and IP rules? The answer depends in part on a much older discussion about global public goods—goods which because of their very nature, the market will fail to supply adequately [71].^8^ Global public goods such as public primary education, the global climate and global health technologies, as well as innovation have positive externalities that will be undervalued by the market. As such, economic theory supports government intervention to promote these goods as a way to correct a market failure [72, 73].

There are special international rules for one particular global public good – intellectual property – which actually requires countries to intervene to protect it. The rules, especially those in the TRIPS agreement, implicitly recognize the need to make it worthwhile to generate new knowledge. The tension in IP policymaking is to balance the need to compensate innovators with the public’s need for access to new ideas and products. Increasingly strict international IP rules, however, do not seem to consider the dangers of over-protection of IP. Indeed, Joseph Stiglitz has shown that “[a]n excessively broad patent system … can raise the price of one of the most vital inputs into the innovative process [knowledge itself] and thus reduce the pace of follow-on innovations'' [72].

Meanwhile, other global public goods do not receive the same protective treatment. Instead, countries must rely on public policy or security exceptions to ensure access to essential products for public health, the environment or other public goods [74]. From an economic perspective, global rules do not provide adequate policy space for governments to address key market failures and should be reformed.

This is not to argue that all government policies toward public goods are “good”, either in terms of achieving their stated purposes or avoiding negative externalities for others. Each government introducing new policies makes decisions based on trade-offs –between global and national interests and between private and public interests. While some pandemic policies earned global support (research and development subsidies for mRNA research), others earned widespread condemnation (export constraints of essential products) and still others received critiques suggesting that the policy would have been better if structured or applied differently (such as public–private innovation partnerships like Moderna-NIH) [23, 75]).

From a scientific perspective, the most effective approach to end the pandemic is at the global level [76]. By its nature the pandemic has crossed (in some places is still crossing) borders repeatedly, fueling wave after wave of infections, illness and death across the globe. However, governance generally occurs at the national level, such that national governments are often the only entities with both the legal authority and practical ability to respond (see [77].^9^ These governments have the responsibility to protect their citizens and residents; and political leaders perceived that their own standing and even tenure would be determined by their success in doing so. By contrast, there is no global “government”, and existing institutions at the supranational level, such as the WHO, lack both the authority to mandate responses by states and the means to deliver sufficient public goods.

### Methodological limitations

There are several methodological and other considerations that should be mentioned when using secondary data from the GTA database.

First, GTA data relies on official government notifications, which is a possible under-reporting bias as this data collection method is contingent on the transparency of governments publishing their policies online. Over-reporting is unlikely however as only measures that are either “implemented or whose future implementation is enacted” [4] are included in the dataset. This present analysis only considered measures for which the date of implementation is available.

We note that the GTA database does not include information about state acts which are likely to be enacted, but still pending in the legislative process. For financial incentives such as subsidies, bailouts and other forms of state aid, an intervention with a volume exceeding USD 10 million is considered meaningful. For interventions targeted exclusively at small and medium enterprises, the volume has to exceed USD 100 million. Further, governments differ in how they announce policies. As noted by the GTA data team, the US government tends to announce each policy separately, while European governments tend to announce policies in bundles.

Moreover, there is likely to be better coverage of nations that are members of the G20 (more generally of countries with larger GDPs), countries whose governments make more information available online, and where traditions of transparent government are strongest. Lastly, the authors have not reviewed the GTA “amber” classifications. Policy interventions are classified “amber” under two circumstances: (a) when the implementation of the policy instrument would likely worsen the relative treatment of some foreign commercial interests or (b) when the implementation of the policy instrument would almost certainly worsen relative treatment of some foreign commercial interest but where no official source can be found to document the measure. For these purposes, an official source refers to a text or online record published by a government body in the implementing jurisdiction, or a text published by an official international organization, such as the WTO [78]. The authors elected to focus on policy interventions classified as “red” for greater certainty but acknowledge that this is almost certainly an under-estimate of the number of policies enacted.

## Conclusions

This research paves the way for many additional studies to flesh out the role of policymaking in a crisis and the impact, potential and actual, of trade and investment rules on those countries making policies. This study could be expanded to include more countries, or additional region-specific studies could be explored to see how countries in the same region have responded to both the crisis and the treaty-based constraints. As the world continues to grapple with the climate crisis over the long term, more research will need to be done exploring how those treaties might constrain climate policymaking or other public policymaking dealing with other types of global crises – like debt or financial stability. This data, and the mapping process of testing for tensions between policymaking and treaty constraints could help to inform many other studies to understand what reforms are needed and how to shape trade and investment treaties in a world battling multiple crises at once.

Countries have deployed many policies aimed at increasing access to essential health products during the pandemic. Many of those policies are in tension with the global trade, investment, and IP rules. This tension must be resolved in favor of governments making policy rather than relying on existing exceptions or pushing national governments to comply more exactly with the rules. Although the pandemic itself does not respect national borders, governance still generally occurs at the national level because national governments are often the only entities with both the legal authority and the practical ability to respond. Presently, for the vast majority of countries, any measure that preferences domestic products, services or investment vis-a vis imports or foreign investors is strongly discouraged, or in some cases prohibited. Any measure that widens the current distribution of production and supply chains could be claimed to “nullify or impair” the benefits that firms expected when their countries signed trade agreements. Our study found that many large and powerful countries were willing to enact policies that directly run up against global trade rules to deal with the crisis, but their measures may still be challenged in the future. Less powerful and smaller economies are even more vulnerable. The global trade regime clearly needs to be re-thought and reformed to allow countries to better address global crises, such as pandemics.

### Supplementary Information

**Additional file 1: Annex.** Mapping of the GTA-identified intervention types onto the domains studied in this paper.

## Data Availability

The datasets generated and analyzed during the current study are available in the Global Trade Alert repository, https://www.globaltradealert.org/data_extraction.

## Abbreviations

BIT: Bilateral investment treaty
CL: Compulsory license
CPTPP: Comprehensive and Progressive Trans-Pacific Partnership
FDI: Foreign direct investment
FTA: Free trade agreement
GATS: General Agreement on Trade in Services (WTO)
GATT: General Agreement on Tariffs and Trade (WTO)
GDP: Gross domestic product
GPA: Government Procurement Agreement (WTO)
GTA: Global Trade Alert
ISDS: Investor-state dispute settlement
LDC: Least developed country
PPE: Personal protective equipment
RCEP: Regional Comprehensive Economic Partnership
SCM: Agreement on Subsidies and Countervailing Measures (WTO)
TRIMs: Agreement on Trade-Related Investment Measures (WTO)
TRIPS: Agreement on Trade-Related Aspects of Intellectual Property (WTO)
USMCA: United States-Mexico-Canada Free Trade Agreement
VL: Voluntary license
WHO: World Health Organization
WTO: World Trade Organization

## Footnotes

1 The reasoning behind the very different treatment of tradable physical goods compared to intellectual property is partly rooted in the concept of innovation as a “public good” and therefore in need of different treatment within the market. A longer discussion of this concept as it applies to both public health and innovation is in the Discussion section.

2 It is important to note at the outset that not every policy implemented by governments during the pandemic effectively and equitably corrected global market failures. The authors do not endorse every government policy cataloged here as “good” or even justifiable.

3 A further comprehensive description of the GTA’s methodology can be found in [3]

4 “Health Sector Interventions” are interventions which impact a combined grouping comprised of nine sectors chosen by the authors: (1) basic organic chemicals, (2) basic inorganic chemicals, not elsewhere categorized (n.e.c.), (3) miscellaneous basic chemical products, (4) pharmaceutical products, (5) chemical products n.e.c, (6) medical & surgical equipment and orthopedic appliances, (7) instruments and control equipment, except optical instruments, (8) petroleum, chemical and pharmaceutical manufacturing services, (9) human health services.

5 South Africa implemented slightly more subsidy measures than trade measures, but the sample size for both South Africa and China are so small as to not be very instructive about trends. “Middle Income” countries are considered to be China, South Africa and India.

6 This refers to domestic processes of complaint by the domestic industry, investigation and findings with respect to the impact on the domestic industry. Although the remedies are governed by domestic law, the SCM Agreement provides guard rails to make sure those processes are fair, transparent and consistent with the general standards governing subsidies (SCM Part V).

7 Moran helpfully lays out the WTO disputes regarding the general exceptions and shows that these are rarely successful but does not conclude that this is problem for states [49].

8 The same economic theory that demonstrates that global public goods will not be adequately supplied by the market in the absence of government policy, also explains why it fails to supply adequate products for poorer economies. “Weak profit incentives discourage commercial research and development investments in diseases of the poor” since the poor have different health priorities than the rich, but do not have the same purchasing power and the priorities of wealthier countries, in terms of both products and innovation, will win out [79]. Although some economists argue that the incentives facing the private sector lead to important efficiency gains, increasing supply and therefore possibility for greater overall distribution [78], studies over the last 30 years have shown that, generally, the private sector under-invests in research and development to address illnesses that plague low-income countries and people because the short-term return on investment is not high [80].

9 In the European Union, some relevant competencies exist at the supra-national EU level.
