# Supplementary material for: Policy responses to COVID-19: lessons for the global trade and investment regime
Source: Global Health. 2023 Sep 1;19:66. doi: 10.1186/s12992-023-00961-6 (PMC10472676; doi:10.1186/s12992-023-00961-6)
Supplement: Supplementary file 1 — Additional file 1: Annex. Mapping of the GTA-identified intervention types onto the domains studied in this paper. [file 12992_2023_961_MOESM1_ESM.docx]

**Annex: Mapping of the** **GTA-identified intervention types onto the domains studied in this paper**

| **Domains** | **GTA-identified intervention types** |
| --- | --- |
| Subsidies | [7] subsidies and state aid |
| Trade measures (tariffs and quantitative restrictions) | [2] export and import measures  [8] trade defense instruments  [9] other instruments |
| Investment measures | [3] foreign investment measures  [5] localization requirements  [1] capital controls and exchange rate policies |
| Public procurement | [6] public procurement |
| IP policies | Not captured in GTA |

*[4] labor force migration rules were not relevant for this paper
